# Inhalation of Carbonic Acid Gas in Dyspnœa

**Published:** 1888-04

**Authors:** 


INHALATION OF CARBONIC ACID GAS IN DYSPNŒA.—Dr. EDWARD WEIL has used inhalations of carbonic acid gas to relieve dyspnœa, and with favorable results. He thinks that the good effect is due to the abolition of reflex sensibility of the pharynx and larynx. The patient inhales the gas for from two to five minutes once or twice a day, in quantities of from two to four litres. When the inhalations are made during dyspnœa this is cut short, and the patient breathes easily and comfortably. When the gas is given between the attacks inspiration becomes more free, and the attacks are prevented, or diminished in frequency, intensity, and duration.—*La Semaine Médicale*, March 7, 1888.

